# Evidence for vibration coding of sliding tactile textures in auditory cortex

**DOI:** 10.3389/fnins.2023.1282566

**Published:** 2023-11-23

**Authors:** Roberta D. Roberts, Aldrin R. Loomes, Hoi Fei Kwok, Alan M. Wing, Harriet A. Allen

**Affiliations:** ^1^Sensory Motor Neuroscience Laboratory, School of Psychology, University of Birmingham, Birmingham, United Kingdom; ^2^School of Psychology, University of Nottingham, University Park, Nottingham, United Kingdom

**Keywords:** texture perception, duplex theory, vibrotactile, brain imaging, somatosensory cortex, auditory cortex

## Abstract

**Introduction:**

Psychophysical studies suggest texture perception is mediated by spatial and vibration codes (duplex theory). Vibration coding, driven by relative motion between digit and stimulus, is involved in the perception of very fine gratings whereas coarse texture perception depends more on spatial coding, which does not require relative motion.

**Methods:**

We examined cortical activation, using functional Magnetic Resonance Imaging associated with fine and coarse tactile spatial gratings applied by sliding or touching (sliding vs. static contact) on the index finger pad.

**Results:**

We found regions, contralateral to the stimulated digit, in BA1 in S1, OP1, OP3, and OP4 in S2, and in auditory cortex, which were significantly more activated by sliding gratings but did not find this pattern in visual cortex. Regions in brain areas activated by vibrotactile stimuli (including auditory cortex) were also modulated by whether or not the gratings moved. In a control study we showed that this contrast persisted when the salience of the static condition was increased by using a double touch.

**Discussion:**

These findings suggest that vibration from sliding touch invokes multisensory cortical mechanisms in tactile processing of roughness. However, we did not find evidence of a separate visual region activated by static touch nor was there a dissociation between cortical response to fine vs. coarse gratings as might have been expected from duplex theory.

## 1 Introduction

The ability to perceive texture through touch plays a vital role in everyday interactions with objects, facilitating, for example, grip control, object identification and sensory appreciation. Contact between a textured surface and the skin causes deformation of the skin which induces stress and strain in the underlying tissue. These changes are sensed by low threshold mechanoreceptors (LTMs). Four types of LTM have been identified in humans and each has a particular frequency response (Johansson et al., 1982; McGlone and Reilly, 2010). Rapidly adapting Pacinian and Meissner corpuscles are sensitive to frequencies in the 20–400 Hz range while Ruffini and Merkel cells have a slowly adapting characteristic and are more sensitive to sustained pressure. Recordings from single cutaneous afferents in rhesus macaque during mechanical scanning of textures over the digit tip show spatial firing patterns can account for the processing of coarse textures with elements of the order of millimeters (Weber et al., 2013). However, information about fine textures with elements of the order of tenths of a millimeter was conveyed through temporal patterns in afferent spiking responses driven by high frequency skin vibrations elicited by sliding of the textures over the digit.

The duplex theory of surface texture perception (Hollins and Risner, 2000; Hollins and Bensmaia, 2007) proposes two distinct forms of coding of LTM information based on spatial and vibration coding. Spatial coding uses information about the spatial distribution of the forces across the skin surface resulting from contact between the skin and the textured surface. Psychophysical evidence suggests perceived tactile roughness through spatial coding is related to the geometry and distribution of the surface elements (Lederman and Taylor, 1972; Lederman, 1974; Morley et al., 1983; Verrillo et al., 1999; Meftah et al., 2000). Spatial coding is thought to be more important in the perception of coarser textures. Vibration coding refers to the use of vibrotactile information generated during sliding movement of the skin over the textured surface. Vibration coding is thought to be more important in fine texture perception in the sub-millimeter range when the spatial resolution of the slowly adapting fibres is exceeded. The contrast between spatial and vibration coding is supported by the finding that discrimination of fine sandpapers and of high frequency spatial gratings is impaired when tactile contact changes from sliding to static, while discrimination of coarse sandpaper or low frequency spatial gratings is unaffected (Hollins and Risner, 2000; Roberts et al., 2020). Moreover, vibrotactile adaptation before tactile testing impairs discrimination of fine but not coarse textures (Hollins et al., 2001).

Is there evidence of a dissociation between cortical regions contributing to spatial and vibration coding of roughness? Human brain imaging studies involving tactile textures have largely focused on which somatosensory areas of the brain are activated by tactile textures in contrast to other touch attributes such as shape, length, softness, orientation (Roland et al., 1998; Servos et al., 2001; Kitada et al., 2006; Allen and Humphreys, 2009). Eck et al. (2016) showed somatosensory and visual cortical activation in roughness judgments based on haptic exploration of dot patterns. The interdot spacing in this task was in the supra-millimeter range, and so may have involved spatial rather than vibration coding. If moving tactile stimuli are assumed to induce vibration, reports of auditory cortex response to moving stimuli applied to the skin of humans and macaques (Foxe et al., 2002; Kayser et al., 2005) might be taken as indirect evidence for vibration coding. This would be seen as complementing findings of auditory cortex response to vibrotactile stimuli in the 20–200 Hz range (Caetano and Jousmaki, 2006; Schurmann et al., 2006; Li Hegner et al., 2007; Nordmark et al., 2012). However, Foxe et al. or Kayser et al. did not directly measure any vibration produced by their stimuli, they did not evaluate the effect of contact dynamics (static versus sliding touch) on auditory responses to touch, nor did they examine overlap of auditory areas responsive to vibration and to sliding stimuli. Therefore, it is unclear whether or not the contrast between auditory cortex activity observed by Foxe et al. and Kayser et al. but absent in the results of Eck et al., should be attributed to the use of vibratory compared with spatial coding predicted by the duplex theory.

The present study uses a region of interest (ROI) approach to examine the processing of texture-related information in the brain in an attempt to identify cortical substrates for the duplex theory of texture perception. This was done by measuring BOLD activation while the index finger of the right hand was touched either a fine or coarse spatial grating. The gratings were either moved across the finger or touched without movement. On the basis of duplex theory we expected that there would be differential activation of vibration sensitive regions (i.e., somatosensory and auditory regions) by sliding compared to static gratings. We also investigated whether there would be differential activation to static gratings in areas likely to be selective for spatial patterns such as the visual cortex.

## 2 Materials and methods

### 2.1 Participants

The study was approved by the University of Birmingham ethics review committee.

Thirteen participants (11 female and all right-handed) aged 18–42 years, mean 25.33, SD 8.97 years took part. There were eleven participants (6 female and 2 left-handed) aged 30 ± 12 (range 18–63) years in the control study. None had any history of conditions affecting the tactile sensitivity of the hands. All were staff and students from the University of Birmingham. All (except 2, in the control study) received cash remuneration for their participation. The participants were told the study was to measure the brain’s activity during touch and were given information about the experimental and MRI procedures. They were able to freely withdraw from the study at any point if they wished to do so.

### 2.2 Materials

**Figure 1:**
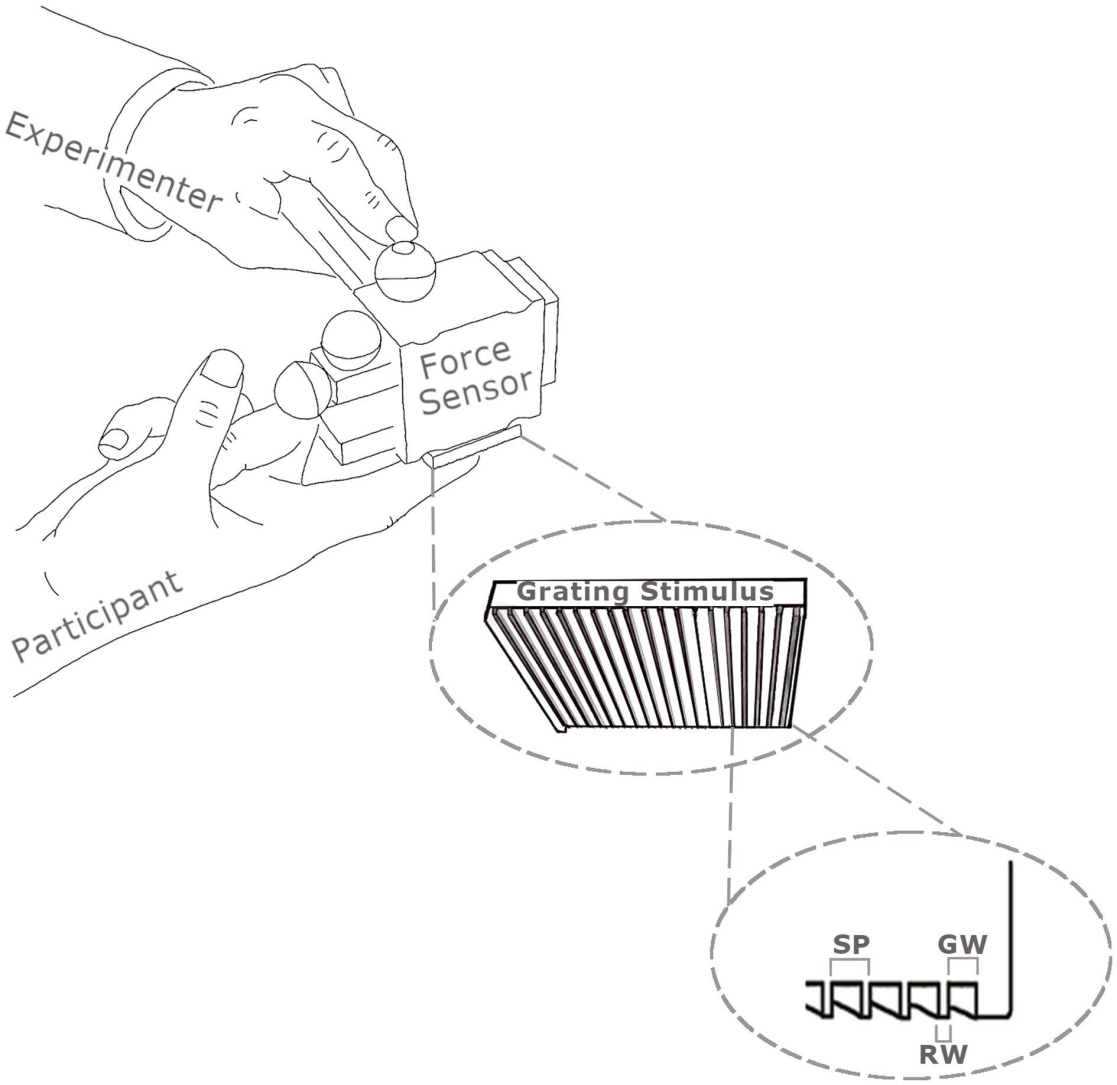
Illustration of the method used to apply the square wave gratings to the distal phalanx of participants’ index fingers using a one degree of freedom force sensor. Normal forces were recorded by the force sensor and movement velocity by infrared cameras tracking reflecting markers (shown as spheres) attached to the force sensor. The spatial periods (SP) of gratings are the sum of the groove width (GW) and the ridge width (RW).

Two imaging studies were run: a main study and a control. For the tactile stimulation in the main study, two rigid polyurethane square wave spatial gratings were used. The gratings were made by cutting Tufset blocks under computer numerical control (CNC). For the main experiment, the fine grating had a spatial period (SP) of 400 μm, a ridge width of 100 μm and groove width of 300 μm. The coarse grating had a spatial period of 1,600 μm, a ridge width of 400 μm and groove width of 1,200 μm. The dimensions of each grating were 30 mm × 36 mm. An example grating and the force sensor used to measure the contact forces when touching participants fingers are shown in Figure 1.

To ensure that the fine grating used was fine enough to engage vibration coding, a preliminary psychophysical test was conducted with 13 participants (11 female, 12 right-handed, mean age 23 ± 5.8). First, using the 400 and 1,600 μm gratings as standards, the individual 71% discrimination thresholds for the fine and coarse gratings in sliding touch conditions were estimated using an adaptive staircase. Mean threshold estimates for the fine and coarse surfaces were 460 ± 92 and 1,050 ± 112 μm, respectively. For each participant a rough and a smooth comparison grating, just slightly rougher, mean 468 ± 91 μm, (in the former case) and smoother, mean 1,018 ± 104 μm, (in the latter case) than that leading to 71% correct discrimination were selected to be compared with the respective standard gratings. An actuated dynamic touch platform (Oddo et al., 2011) was used to control the contact force at 0.6 N and compare the performance under static and sliding (20 mm/s) touch conditions. One sample t-tests were used to compare performance in each movement x texture condition to the 71% correct threshold. Alpha was 0.013 following Bonferroni correction. The participants were significantly worse than the 71% correct threshold at discriminating the rougher of a pair of fine gratings under conditions of static contact, *t*(12) = −3.85, *p* = 0.002, d = −1.067 (CI: −1.74, −0.34), (percent correct 56.4 ± 13.7%). In contrast, performance for the sliding fine and both the sliding and static coarse gratings were no different from the threshold. This finding confirms that, in a discrimination task, the fine grating but not the coarse grating, benefits from sliding contact consistent with duplex theory (see Roberts et al., 2020 for similar findings with a constant-stimulus design using similar square wave gratings).

In the control study four rigid polyurethane square wave spatial gratings were used. Two from the main study (SP 400 and 1,600 μm), as well as two of slightly different spatial period (480 and 1,280 μm) for use in an oddball detection task.

### 2.3 fMRI design

**Figure 2:**
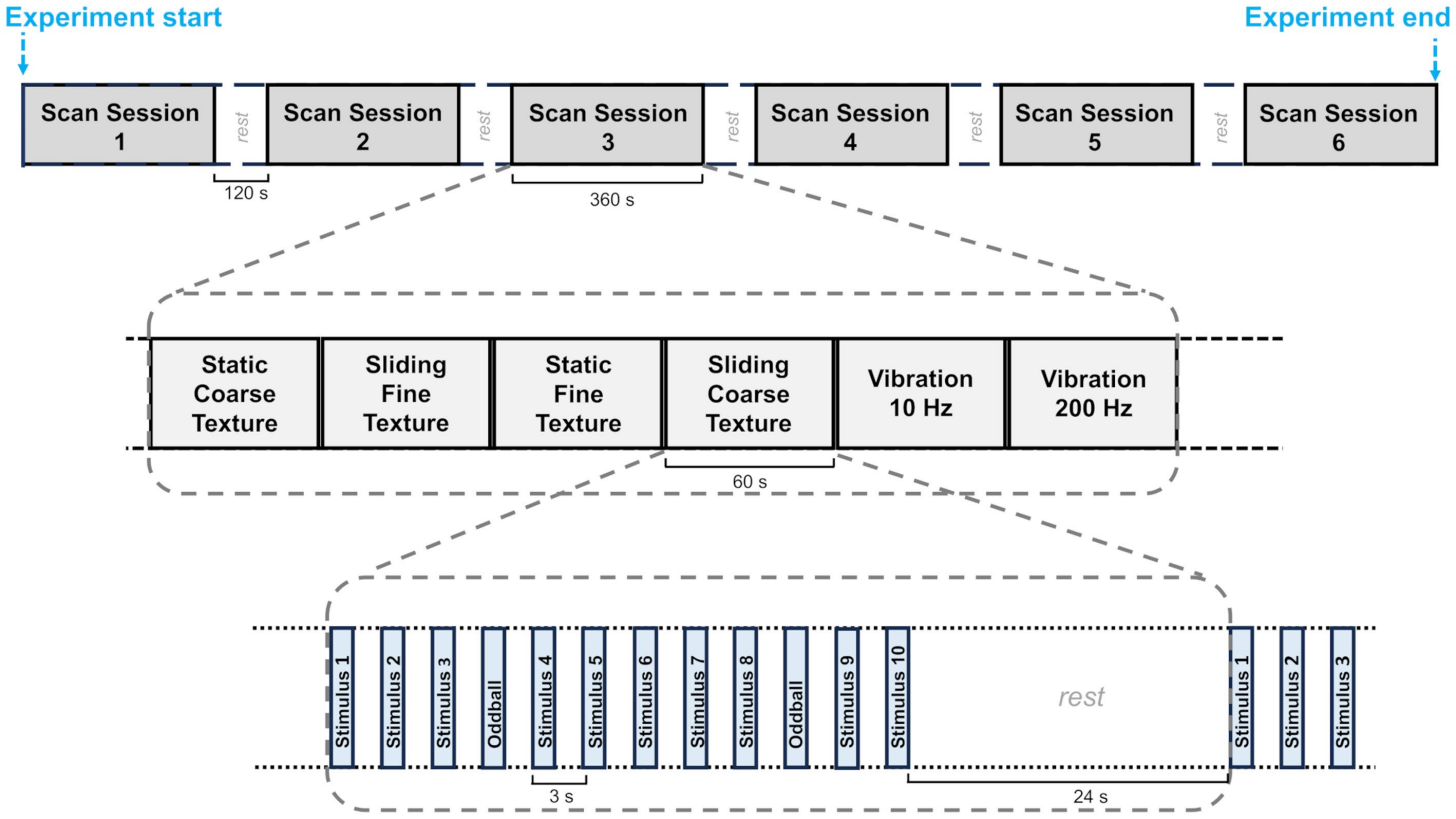
A diagram showing the fMRI block design. Each participant was scanned in an experiment made up of 6 scan sessions each lasting 6 min and separated by a 120 s rest period. These sessions are shown at the top of the figure. In each scanning session the six experimental conditions were presented one after the other. The order of the conditions was randomised. Twelve trials, each with stimuli lasting 1.5 s, were presented in each condition. The trials were followed by a 24 s rest period before the following block of 12 trials began.

A block design was used for the main study (see Figure 2). Each experiment consisted of 6 scan sessions, each lasting 6 min and containing all of the possible conditions. There was a 120 s break between the scan sessions for each experimental session. There were six conditions composed of 2 gratings (fine gratings – SP 400 μm vs. coarse gratings SP 1600 μm), combined with 2 movement conditions (sliding vs. static) plus two vibrotactile stimuli (10 Hz and 200 Hz) presented on the skin and used as localisers for vibration sensitive regions (see below). Within each scan each condition was presented for one block of 36 s with 24 s rest intervals between each condition/block. Each block consisted of twelve 3 s trials (including oddball trials). The order of experimental conditions within a scan was randomised across the six sessions.

In the control study, there were four conditions comprising 2 gratings (fine vs. coarse gratings with spatial period of 480 or 500 μm vs. 1,280 or 1,600 μm), combined with 2 movement conditions (sliding vs. static). The experiment consisted of 6 scan sessions. Each scan lasted 240 s and comprised 4 blocks of 12 trials (36 s) with a 24 s break between blocks. Each experimental condition occurred once within a block and the order of presentation was randomised across blocks. There was a 195 s break between scans.

### 2.4 Stimulus presentation and task

The participants wore earplugs and headphones and kept their eyes closed. An experimenter, standing next to the participant’s right hand and just outside the scanner bore, delivered the tactile and vibrotactile stimulation in response to an auditory cue. In the main study on static touch trials, the experimenter touched the participant’s right index finger with the grating for 1.5 s with a normal force target of 0.6 N and without any lateral movement across the fingertip. On sliding touch trials, the experimenter moved the grating across the participant’s index fingertip from right to left with target speed of 20 mm/s and target normal force of 0.6 N for 1.5 s. The experimenter received prior training with practice so that stimulus force and velocity were as constant as possible and extraneous movements were minimised. The force and velocity were measured using a custom-made MR-compatible optical force transducer connected to the computer via a USB-6009 DAQ system and MR-compatible Proreflex motion tracking system (Qualisys, Sweden) respectively during the scan. On vibrotactile trials, a 10 Hz or 200 Hz vibrotactile stimulus was delivered to the participant’s right index finger for 1.5 s. The MR compatible vibrotactile stimulator (Quaerosys Piezostimulator)^1^ was manually positioned against the participant’s fingertip by the experimenter.

The experiments were made up of stimulation and odd-ball trials. In the stimulation trials the textured surfaces were applied to the palmar skin on the pad of the index finger. There were 0–3 oddball trials in each block, included to keep attention focused on the hand. In the main study, during oddball trials the tactile stimuli were applied to the participant’s thenar eminence (the skin on the palm of the hand, at the base of the thumb) instead of the index finger pad. The participant was required to count the oddball trials and report at the end of each scan.

In the main study the static and sliding trials were matched in target duration and normal force. However, it might be argued that the sliding stimulus was more salient and might have attracted more attention than a single sustained contact of the static stimulus. Thus, in the control study, the following changes were made. In static trials the stimulus touched the index finger twice (contact- lift- replace - lift) while in sliding trials it was stroked in proximal-to-distal direction across the finger pad. The duration of application (including lift and replace time in the double touch) was 1.5 s. In stimulation trials in the control study the stimulus was the greater SP grating (500 or 1,600 μm) for the fine and coarse conditions, respectively. In oddball trials the stimulus was the lesser SP grating (480 and 1,280 μm). Within a block, oddball trials, included to focus attention on grating spatial period, only occurred after at least two regular trials. There were 2–4 oddball trials in each block. Participants reported the number of oddball stimuli on completion of each scan.

### 2.5 Vibrotactile localiser

Vibrotactile localisers consisted of 4 blocks of a vibrotactile stimulus placed on the right index finger. Two of the blocks had a 10 Hz stimulus, and the other two a 200 Hz stimulus. The blocks were interleaved and presented in a single session. Participants were asked to count the number of times a 66 Hz oddball vibration was presented. Oddballs could occur on any trial in the block after the first 2 regular trials were completed. Between 2 and 4 oddballs were presented in each block.

Each vibrotactile localiser trial lasted 3 s (1.5 s stimulus application +1.5 s rest) with 12 trials presented in each block. Each 36 s of stimulation in a block was followed by a 24 s rest period before the next block commenced.

### 2.6 Imaging

The study was conducted in a 3 T Philips Achieva scanner. The functional scans used an echo planar imaging (EPI) sequence (TR = 3 s, TE =35 s, flip angle = 85°, SENSE factor = 2). It consisted of 48 slices and there were 120 volumes in each scan. The voxel size was 2.5 mm × 2.5 mm × 2.5 mm. A T1-weighted anatomical scan was conducted in the same session for each participant with 1 mm^3^ resolution.

### 2.7 Data analysis

The contact forces and movements used when applying the stimuli to the skin were analyzed using Matlab (The MathWorks, Inc.). The MRI data were processed and analyzed using FSL (FMRIB software library).^2^ The following pre-statistics processing was applied; motion correction using MCFLIRT (Jenkinson et al., 2002); slice-timing correction using Fourier-space time-series phase-shifting; non-brain removal using BET (Smith, 2002); spatial smoothing using a Gaussian kernel of FWHM 5 mm; grand-mean intensity normalisation of the entire 4D dataset by a single multiplicative factor; high-pass temporal filtering (Gaussian-weighted least-squares straight line fitting, with sigma = 42.0 s). Time-series statistical analysis was carried out using FILM with local autocorrelation correction (Woolrich et al., 2001). Higher-level analysis (mixed effects) was carried out using FLAME (FMRIB’s Local Analysis of Mixed Effects) stage 1 (Beckmann et al., 2003; Woolrich et al., 2004; Woolrich, 2008). The contrasts included each condition against baseline (no touch). To find effects of the presence of movement as well as the texture variable, ROI analysis was conducted in functional defined vibrotactile regions and anatomically defined somatosensory regions as well as comparisons between conditions as described below. Z (Gaussianised T/F) statistic images were thresholded using clusters determined by Z > 2.0 and a (corrected) cluster significance threshold of *p* = 0.05 (Worsley, 2001), unless otherwise stated below.

In addition, an ROI mask was created using coordinates relating to Heschl’s Gyrus for the control experiment. This was chosen based on previous studies identifying selectivity to auditory stimulation in this area (Reiner et al., 2011; Man et al., 2015). ROI masks were created using the Harvard Oxford Atlas in FSLeyes and applying this to each individual’s anatomical T1 scan. Analysis was then performed to identify peak activation in both the left and right hemispheres during static and sliding tactile stimulation.

## 3 Results

### 3.1 Main study

The behavioural oddball data of 5 participants were lost. The remaining participants reported the correct number of odd balls in the majority of blocks of trials (≥75% of trials). When errors occurred they involved under-reporting of oddball stimuli (mean error was 1, SD = 1.1).

#### 3.1.1 Force and velocity

The mean velocities when coarse and fine gratings made contact with the skin were 25.4 ± 8.1 mm/s and 26.8 ± 8.8 mm/s, respectively. This difference was not statistically significant. The mean contact duration for all six conditions ranged from 1.05 ± 0.23 s to 1.21 ± 0.28 s and the differences were not statistically significant.

**Figure 3:**
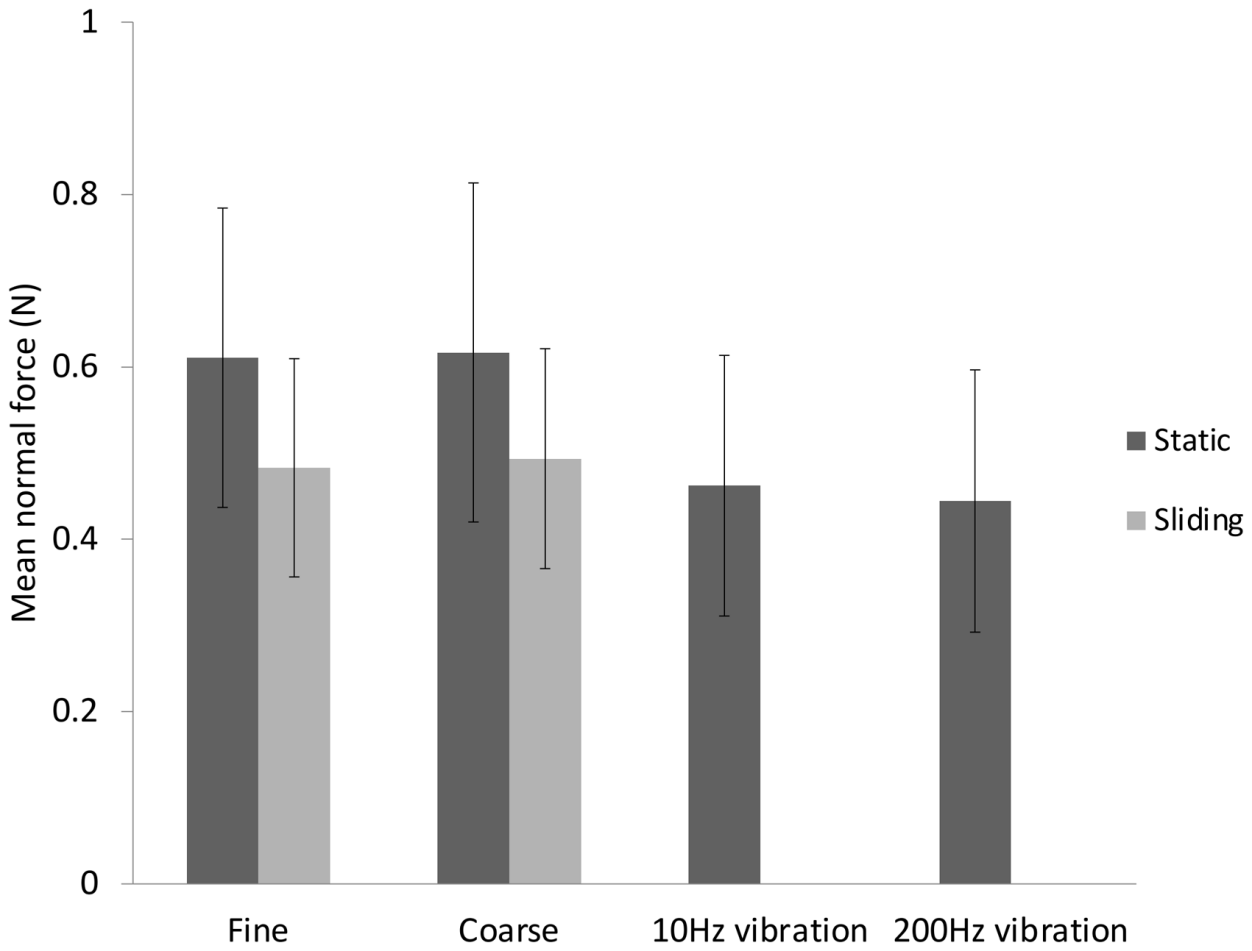
Mean contact force ± standard deviations for the six conditions in the main study.

The mean contact force was 0.52 ± 0.17 N and was slightly higher in static touch (0.61 ± 0.18 N) than sliding touch (0.49 ± 0.12 N) (Two-way ANOVA, *F*(1,12) = 8.09, *p* = 0.007, $\eta_{p}^{2}$ = 0.68) (see Figure 3). The mean contact forces in the two vibrotactile conditions were no different from each other or from the sliding touch conditions. Vibrations were applied with lower mean contact force (0.45 ± 0.15 N) than static touch forces, *t*(12) = −5.09, *p* < 0.001, d = −1.41 (CI: −2.18, −0.62).

#### 3.1.2 Imaging

**Figure 4:**
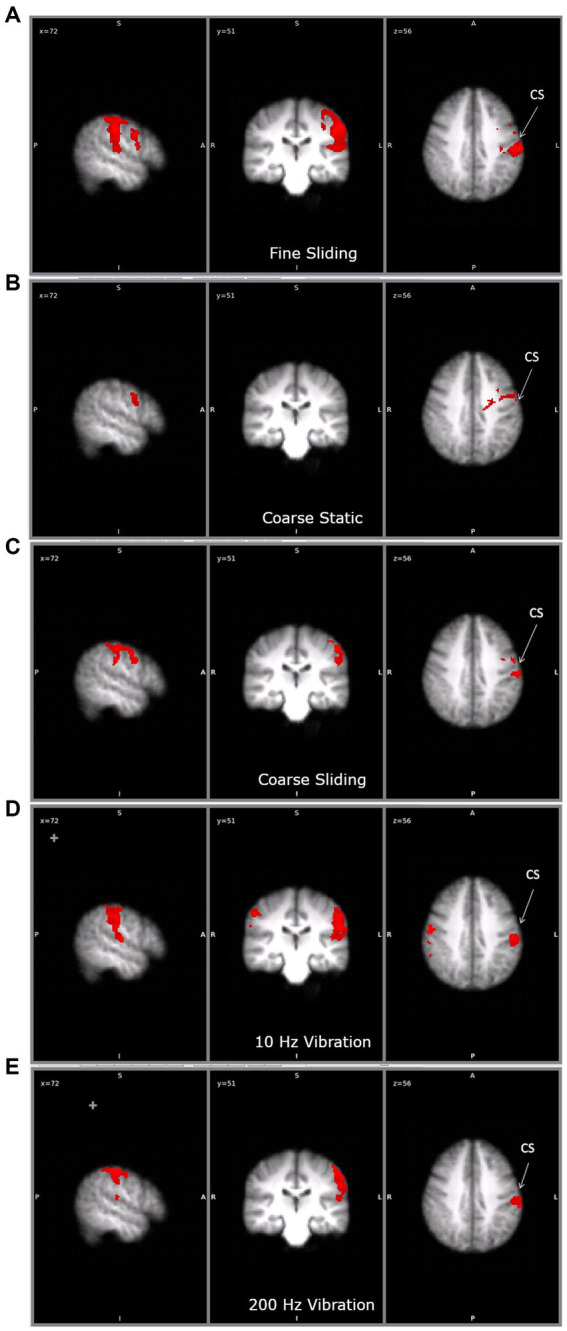
The clusters of activation during **(A)** fine sliding **(B)** coarse static **(C)** coarse sliding **(D)** 10Hz vibration and **(E)** 200 Hz vibration stimulation in the main study. CS: central sulcus. For each condition, the activation is shown on the same three slices, to allow comparisons. Right hand panel shows left hemisphere. On the middle panel the left hemisphere is shown on the right (radiological convention).

To ensure our ROI analyses captured the overall pattern of brain activity, a whole brain analysis was performed. The activation for each condition compared to baseline (no touch) is shown in Figure 4 and Table 1. No region of the brain was more active than baseline in the fine static condition. Sliding touch by fine (Figure 4A) and coarse gratings (Figure 4C) resulted in clusters centred at the postcentral gyrus and extended to the supramarginal gyrus. Static touch by coarse gratings (Figure 4B) resulted in a small cluster of activation centred at the contralateral precentral gyrus. For both vibrotactile conditions (Figures 4D,E), there were activations centred at the anterior division of supramarginal gyrus contra-lateral to the stimulation, extending to Post-Central Gyrus and Heschl’s gyrus. There was also additional ipsilateral activation in the 10 Hz vibrotactile condition.

**Table 1:** The coordinates of the maximum z of the clusters identified by comparing the tactile and vibrotactile conditions with the baseline.

Condition	Voxels	z-max	X	Y	Z	Anatomical label
Fine sliding	5,036	4.97	−54	−24	40	L Postcentral gyrus
Coarse static	844	3.63	−38	−4	30	L Precentral gyrus
Coarse sliding	1,387	4.78	−60	−20	36	L Postcentral gyrus
10 Hz vibrotactile	1,242	4.58	−58	−22	26	L Postcentral gyrus
						Supramarginal gyrus, anterior division
	786	2.87	56	−28	48	R Supramarginal gyrus
200 Hz vibrotactile	1,091	4.51	−54	−28	52	L Postcentral gyrus

The anatomical labels were based on the Harvard-Oxford cortical structural atlas with 20% as the threshold.

##### 3.1.2.1 Activation to touch stimulation in vibration-selective regions

On the basis of duplex theory we expected activation in vibration sensitive areas for sliding but not for static gratings. To test this we restricted our analysis to those regions that responded to the 10 Hz and 200 Hz vibrational stimuli. There was no significant difference in activation between the two vibration stimuli so, to get a robust and inclusive ROI for vibration-sensitive regions, we combined the two conditions and limited analysis to within the resulting mask (uncorrected *p* < 0.05). Conjunction analysis was conducted according to a previously described method (Nichols et al., 2005) to explore the areas shared between those activated by the gratings and those activated by vibrotactile stimuli. Only sliding gratings activated areas sensitive to vibrotactile stimulation (Table 2). There was no overlap between areas activated by static gratings and areas activated by vibrotactile stimulation.

**Table 2:** The number of voxels and coordinates and anatomical labels of the maximum z for the clusters resulting from conjunction between fine and coarse sliding gratings stimulation and vibrotactile stimulation using a cluster z threshold of 2.0 and *p* value of 0.05.

	Voxels	*p*	z-max	X	Y	Z
Fine sliding ⋂ vibrotactile	2024	0.000229	3.94	74	52	53
Coarse sliding ⋂ vibrotactile	1,222	0.0323	3.97	74	52	52

##### 3.1.2.2 Differential activation for different touch stimuli

On the basis of duplex theory we predicted differential activation for coarse and fine stimuli in somatosensory regions. We defined anatomical regions of interest including Brodmann Areas (BA) BA1, BA2, BA3a, BA3b of contralateral somatosensory areas of S1, OP1, OP2, OP3 and OP4 of contralateral S2. This restriction reflects previous findings, in both humans and non-human primates, of activation of the primary (Chen et al., 2001; Friedman et al., 2004) and secondary somatosensory (Jiang et al., 1997) cortices in humans during contact with textured and vibrating surfaces. In addition, we included TE1.0, TE1.1 and TE1.2 of contralateral primary auditory cortex (based on the Juelich Histological Atlas with threshold of 0.2 applied to the probabilistic map, partially corresponding to Heschel’s Gyrus see Warrier et al., 2009), following findings of activation in the primary auditory cortex when vibrotactile stimuli were applied to the skin (Caetano and Jousmaki, 2006; Schurmann et al., 2006). We also examined whether there were activations in visual areas (V1) and frontal eye field which previously were suggested to be involved in tactile spatial processing (Zhang et al., 2005). The frontal eye field ROI was delineated using WFU_PickAtlas (Maldjian et al., 2003). Only some of the somatosensory cortex is sensitive to vibrotactile stimulation so Table 3 shows areas of activation for our different conditions, with and without masking by vibrotactile specific regions as above.

**Table 3:** The size, maximum z-value and the coordinates of the voxel with the maximum z value of the clusters identified to be significantly activated in the four grating conditions compared to baseline, with or without vibrotactile area mask.

		Without vibrotactile mask					With vibrotactile mask				
Condition	Area	No. voxels	z-max	z-max			No. voxels	z-max	z-max		
				X	Y	Z			X	Y	Z
Fine static	BA1	178	3.12	−56	−26	52	177	3.12	−58	−22	42
	BA2	319	3.32	−46	−42	60	242	3.32	−46	−42	60
	BA3b						27	2.58	−48	−14	50
Fine sliding	BA1	1,245	4.25	−54	−22	40	652	4.88	−54	−22	40
	BA2	1,331	4.97	−54	−24	40	624	4.97	−54	−24	40
	BA3b	709	4.25	−46	−20	56	158	3.99	−46	−18	56
	OP1	727	4.56	−60	−18	26	587	4.56	−60	−18	26
	OP3						21	3.65	−46	−20	16
	OP4	377	4.79	−60	−18	32	313	4.79	−60	−18	32
	TE1.0	154	3.81	−50	−22	16	119	3.81	−50	−22	16
	TE1.2						20	3.16	−56	−18	10
Coarse static	BA1	213	3.33	−56	−22	50	210	3.33	−56	−22	50
	BA2	519	3.40	−58	−36	38	295	3.33	−56	−22	50
	BA3b						28	3.00	−50	−22	44
Coarse sliding	BA1	675	4.78	−56	−22	50	210	4.78	−56	−22	50
	BA2	979	4.78	−60	−20	36	412	4.78	−60	−20	36
	BA3b	178	4.08	−52	−20	52	110	4.08	−52	−20	52
	OP1	189	3.69	−60	−22	32	189	3.69	−60	−22	32
	OP4	153	4.35	−62	−18	34	153	4.35	−62	−18	34

The cluster z-threshold was 2.0 with *p* < 0.05, in addition, for the masked analysis (where the number of comparisons is lower) we report only those clusters where *k* ≥ 20.

During static touch by fine and coarse gratings, there were significant activations in BA1 and BA2 but no activations could be found in secondary somatosensory areas and primary auditory cortex. During sliding touch by both fine and coarse gratings, significant activations were found in BA1, BA2, BA3b, OP1 and OP4. Restricting our analysis to only those sub areas specific to vibrotactile stimuli we found additional activations in BA3b (static coarse and fine), OP3 (sliding fine), TE1.0 and TE1.2 (sliding fine). No activation was found in somatosensory regions BA3a, OP2, auditory regions TE1.1 and the visual areas for any condition.

**Figure 5:**
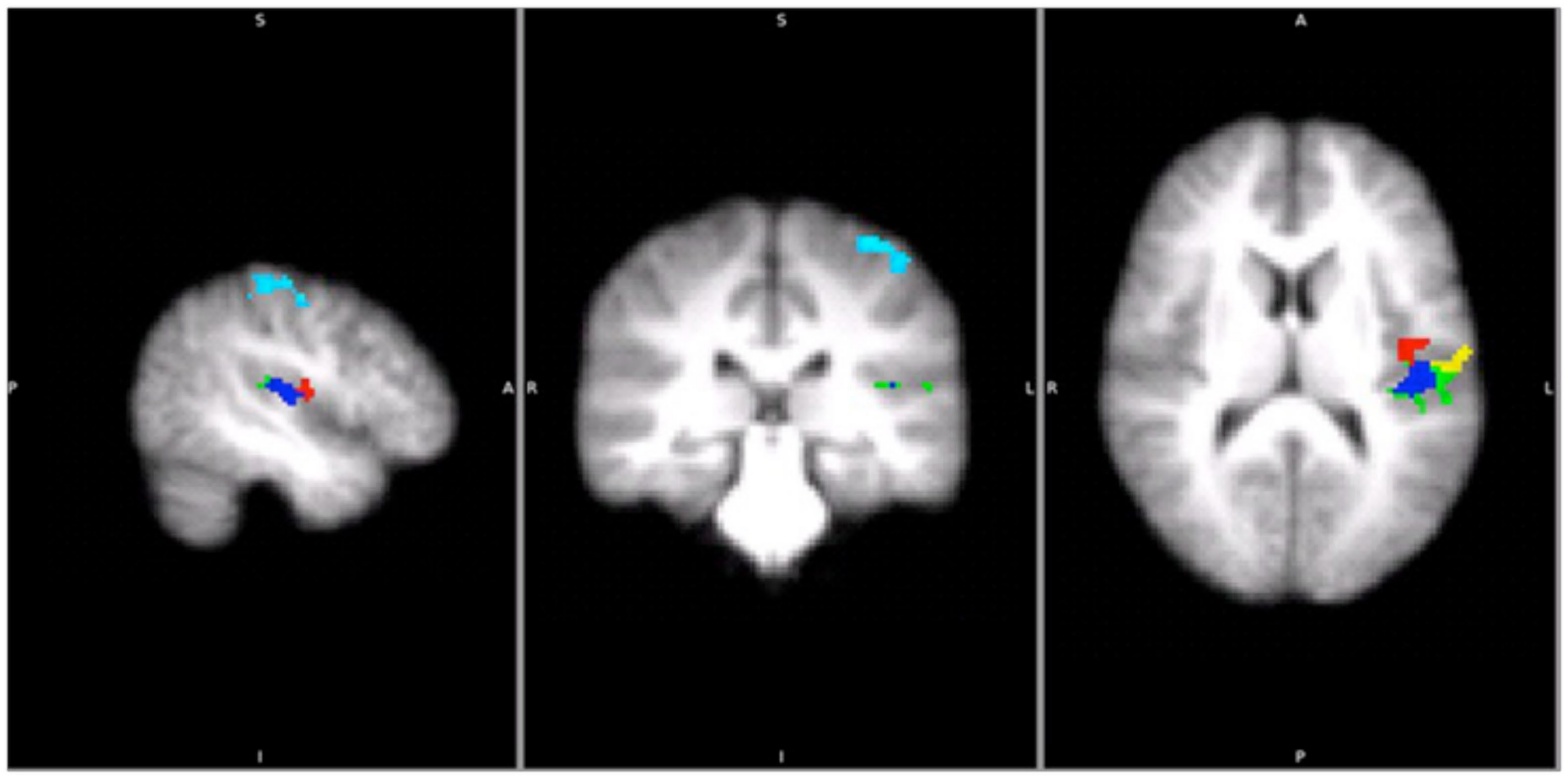
Areas that were significantly activated by sliding gratings (using two-way ANOVAs *p* < 0.05 with cluster z threshold of 2.0) in the main study. Light blue: BA1, Green: OP1, Red: OP3, Yellow: OP4 and Blue: TE1.0.

We were interested to test whether our ROIs (excluding BA3a, OP2 and TE1.1, see above) showed differential activation to coarse vs. fine or static vs. sliding stimuli. Two-way ANOVAs with texture (coarse vs. fine) and movement (static vs. sliding) as factors were conducted on the activation in the individual sub-divisions of primary and secondary somatosensory areas, and the primary auditory cortex with and without masking by vibrotactile areas. Clusters were significantly more active in sliding conditions in BA1, OP1, OP3, OP4 and TE1.0 (Table 4; Figure 5) and additionally in vibrotactile regions of BA3b. No cluster was found within these anatomical areas to have activation significantly affected by the type of texture. No cluster was found to have activation significantly affected by the interaction between texture and movement. Paired t-tests did not reveal any significant differences between the fine sliding and coarse sliding conditions within these areas.

**Table 4:** Somatosensory and vibration-sensitive regions significantly modulated by whether or not the texture moved.

	Area	ROI size (voxels)	Voxels	*p*	z-max	z-max X	z-max Y	z-max Z
						(mm)	(mm)	(mm)
Without vibration mask	BA1	2078	315	0.02	3.02	−50	−12	52
	BA3b	2,256	No cluster identified					
	OP1	1,294	242	0.02	3.13	−56	−20	12
	OP3	518	73	0.05	2.85	−42	−16	12
	OP4	1,062	149	0.04	3.13	−56	−20	12
	TE1.0	469	148	0.02	2.92	−52	−24	14
With vibration mask	BA1	698	111	0.04	3.02	−50	−12	52
	BA3b	187	20	0.04	2.90	−50	−12	50
	OP1	662	160	0.02	3.13	−56	−20	12
	OP4	399	81	0.03	3.13	−56	−20	12
	TE1.0	150	84	0.01	2.92	−52	−24	14

Statistics as above.

### 3.2 Control study

It is possible that a single static contact results in a less salient touch stimulus than a sliding contact. To control for this a second study was run in which the static condition was modified to include a double static touch. The aim was to determine whether a contrast between sliding and static touch would be maintained with increased static touch saliency. In a further change, the oddball task was altered to require attention to roughness and vibrotactile frequency rather than touched location.

The behavioural data suggested oddball detection was more difficult in the control than in the main study. On average, reports of oddballs in test sessions deviated from the actual number of oddballs by 4, with a standard deviation of 2.7 oddballs.

**Figure 6:**
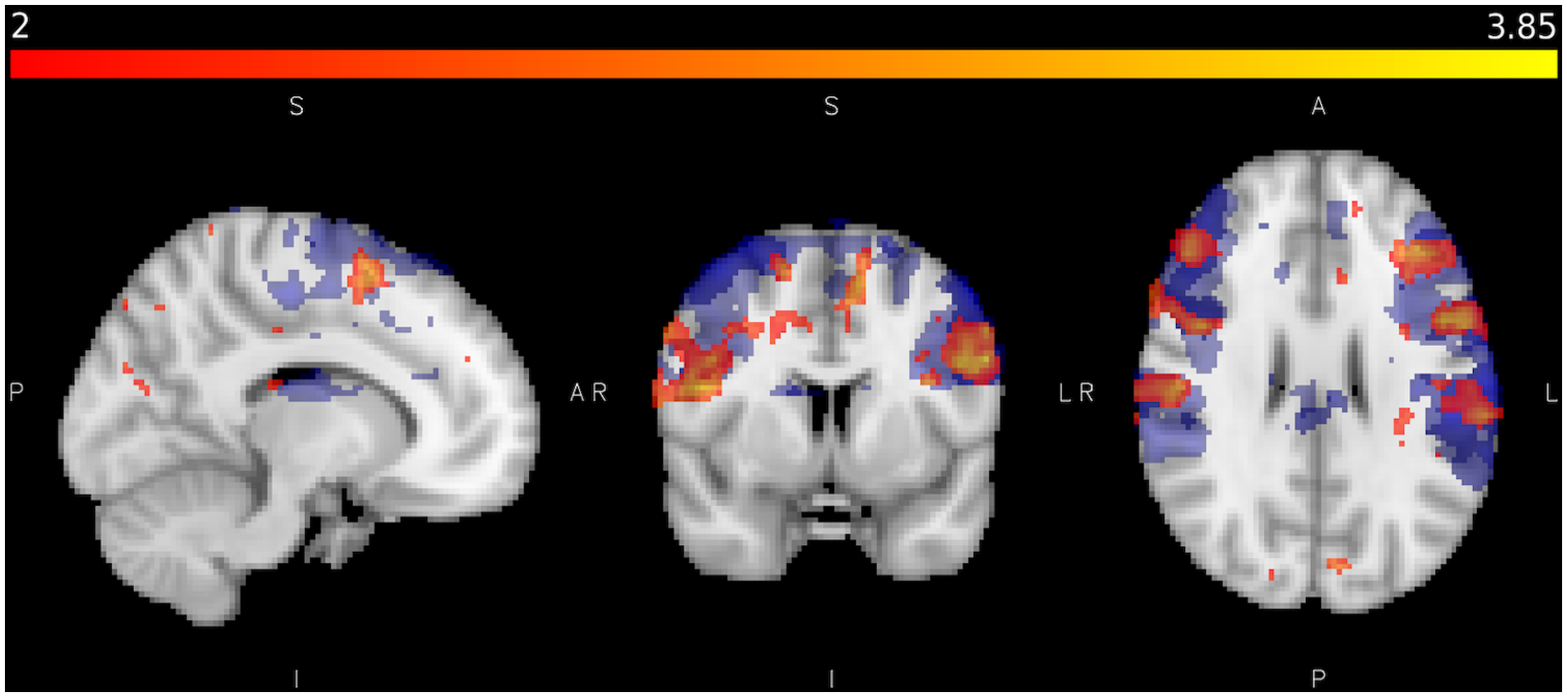
Shows areas activated by sliding touch when activation from static touch is subtracted (red/yellow) and areas activated by vibration (blue) where Z < 2.0, *p* < 0.05 in the control study. Colour bar represents Z statistics between 2 and 3.8.

**Figure 7:**
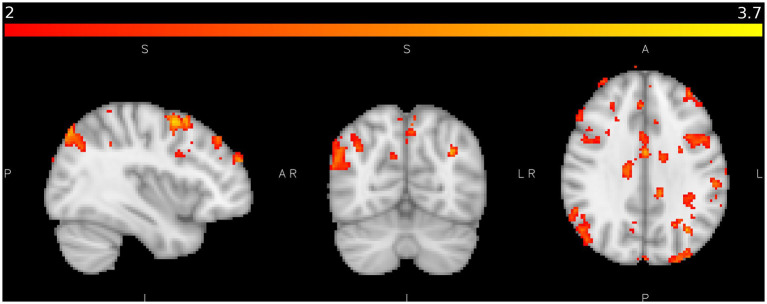
shows areas of significant activation in the control study during coarse texture tactile stimulation when activation from fine texture tactile stimulation is subtracted where Z > 2.0, *p* < 0.05. Colour bar as Figure 4.

The analysis of activation over the whole brain showed that there was significant activation for sliding but not static gratings in areas selective for vibration in these data (Table 5; Figure 6). Replicating the main study, we found a difference between fine and coarse stimuli in somatosensory regions (Table 5; Figure 7). This suggests the difference between the static and sliding stimuli is not due to the task or salience of the stimuli (see discussion).

**Table 5:** The coordinates of the maximum z of the clusters identified during sliding stimulation and coarse texture stimulation in the control experiment.

Contrast	Voxels	*p*	z-max	MNI Coordinates			Anatomical label
				X	Y	Z	
Sliding > Static	4,880	2.35e-08	3.85	50	−30	52	R Postcentral gyrus, R Anterior supramarginal gyrus
	3,922	4.17e-07	3.73	−56	−26	30	L Anterior supramarginal gyrus, L Postcentral gyrus
Coarse > Fine	3,354	1.01e-06	3.70	26	30	52	R Superior frontal gyrus
	978	0.0182	3.41	58	−58	26	R Angular gyrus, R Superior lateral occipital cortex

The anatomical labels were based on the Harvard-Oxford cortical structural atlas with 20% as the threshold.

**Figure 8:**
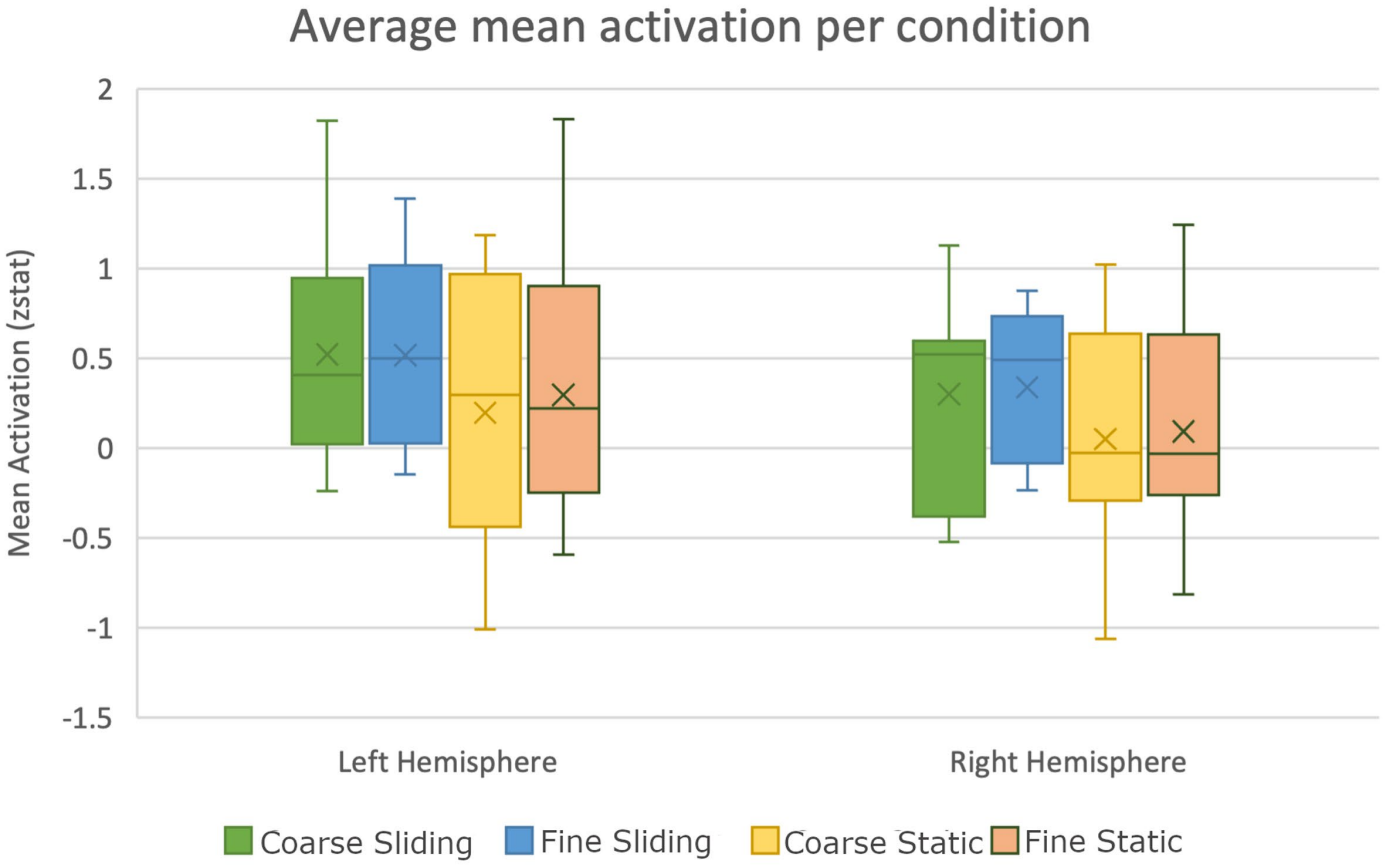
Box plot showing significantly higher activation of auditory areas during sliding texture perception compared to static stimulation in the control study (error bars show standard error).

We also investigated activation to touch in auditory areas in the control study. Heschl’s Gyrus was defined as an area of the Primary Auditory cortex located bilaterally within the Sylvian fissure (Brodmann areas 41 and 42). Mean activation was calculated during each tactile stimulation condition. An ANOVA was conducted using the peak activation in Heschl’s gyrus (defined by Z > 3.2, *p* < 0.05). There were reliable main effects of hemisphere (*F*(1,10), = 14.01, *p* = 0.004, $\eta_{p}^{2}$ = 0.58) and movement type (*F*(1,10) = 5.26, *p* = 0.045. = 0.68, $\eta_{p}^{2}$ = 0.35). No significant interactions were observed. Figure 8 shows mean peak areas of activation in Heschl’s Gyrus compared to baseline across participants. Greater activation is evident in the left hemisphere in all conditions. Significantly greater activation was observed in Heschl’s Gyrus with sliding tactile stimuli. This replicates the finding of activation relating to sliding touch in auditory brain regions, with a stronger static stimulus intensity, different task and different participant group.

## 4 Discussion

Psychophysical research on roughness perception has highlighted the importance of vibration cues for the perception of fine textures contrasting with spatial cues which are sufficient for the perception of coarse textures (Hollins and Bensmaia, 2007). Vibration cues arise from sliding contact between the skin and textured surfaces while spatial cues result from static contacts. In this paper we sought evidence of a contrast between brain activation associated with sliding versus static touch using fine (400–500 μm) versus coarse (1200–1,600 μm) spatial gratings. An experimenter applied the gratings to the skin of the participants lying in the MRI scanner. Based on findings of auditory cortex activation with vibrotactile stimulation (Nordmark et al., 2012) and of auditory activation with stimuli moving across the skin (Foxe et al., 2002; Kayser et al., 2005), we expected auditory cortex activation in the case of sliding but not static contact. In our main study the static contact was maintained for 1.5 s. In a follow-up study, static contact was applied, then released and re-established halfway through the 1.5 s period of stimulation. This was used as a control for the possibility that adaption effects in the main study had resulted in lower intensity for static touch conditions due to the single, continuous period of contact.

The texture related activity we found in primary and secondary somatosensory cortices was broadly consistent with that previously reported for tactile stimulation of the hand. Previous studies examining areas activated by touched textures have found activity in a number of areas in both primary and secondary somatosensory cortices. In our experiment, spatial gratings applied to the index finger generated activations in primary somatosensory areas BA1, BA2 and BA3b, corroborating previous reports of texture related activations in S1 in non-human primates (BA1 and BA3b: Sinclair and Burton, 1988; Sinclair and Burton, 1991) as well as in humans (BA2, BA1, BA3b: Bodegard et al., 2001; Carey et al., 2008). Interestingly, while our static gratings were applied with slightly higher contact force than were the sliding gratings, we found that S1 activation by the sliding gratings was greater than that with static gratings.

Our findings for the secondary somatosensory area were also generally consistent with reports of texture related activity in that region. Of the four subdivisions of S2 (OP1, OP2, OP3 and OP4), tactile stimulation of the hand has previously been found to generate clusters of contralateral activations at the border between OP1 and OP4 and extending to OP3 (Eickhoff et al., 2007). Moreover, greater activation has been reported in OP1 when the skin was touched by coarse compared with fine textures (Simoes-Franklin et al., 2011). While our study revealed texture related activity in OP1 and OP4, this activity was restricted to sliding textures, with no differentiation by surface roughness. However, we failed to replicate previous reports of bilateral S2 activity for tactile stimuli (Kitada et al., 2005; Li Hegner et al., 2007). We were only able to find activation of ipsilateral S2 in our uncorrected images (value of *p* = 0.05 uncorrected). It is worth noting that Eickhoff et al. had to adopt a relatively lenient threshold to find significant ipsilateral S2 activity for tactile stimuli. The discrepancies in the findings across different studies, including our own, may be due to task differences. Evidence for an effect of task on patterns of activation in response to textures making contact with skin has been shown by Kitada et al. (2005). Participants in their study were required to produce magnitude estimates when touching square wave gratings in some trials. In other trials, the participant were instructed to simply attend to the stimuli. Activation patterns common to both tasks were found in S1, parietal operculum and insula. However, roughness-related activation in parietal operculum and insula was only observed with a roughness estimation task. The participants of Li Hegner et al. (2007), where bilateral S2 activity was also reported, were engaged in a roughness discrimination task. In contrast, there was no requirement to discriminate the nature of the tactile stimulus in our main study, or in the study of Eickhoff et al. Furthermore, activity in the secondary somatosensory cortex has been found to vary with attentional demand (Burton et al., 1999). It is possible that our main task may not have required sufficient attention to generate an ipsilateral response in S2. In our control study we used a variation on the oddball detection task (oddballs were defined by a frequency/texture rather than spatial location difference from the standard stimuli) and, under those conditions, we did find activation in S2 for all texture conditions. Neural responses to textured surfaces have also been found to be affected by the mode of touch with S1 regions showing greater activation with actively touched than passively felt stimuli (Simoes-Franklin et al., 2011). Like most work examining the neural mechanisms underpinning texture perception, the present study applied textured stimuli to passive participants. Given that touching, especially touching textures, is primarily an active process it would be interesting in future work to examine how brain regions implicated in different aspects of texture perception are affected by the mode of touch and the resulting forces and kinematics.

We also examined the overlap between those areas activated by vibrotactile stimuli and by our various texture conditions. Cortical regions responsive to vibration were localised by applying vibrotactile stimuli to the hand. The vibrotactile stimuli elicited activation in auditory cortex. With auditory stimuli, changes in frequency of stimulation move activation location in auditory cortex in tonotopic manner (Brewer and Barton, 2016). In the present study, we observed no clear changes in location of activation with 10 Hz versus 200 Hz vibrotactile stimulation, nor was there any difference in level of activation between the two frequency conditions. We found significant overlap between regions activated by vibrotactile stimuli and by sliding gratings, regardless of whether the gratings were fine or coarse. Importantly, the activation for sliding gratings included regions in the auditory cortex.

Hollins and colleagues’ psychophysical studies (Hollins and Risner, 2000; Hollins et al., 2001, 2006; Hollins and Bensmaia, 2007) were primarily directed at the duplex theory of texture perception and strongly suggested that vibration, generated by the relative motion between the skin and a sliding surface (Bensmaïa and Hollins, 2003), plays a significant role in the perception of sliding textures. In particular, it was suggested that vibration cues are critical for the perception of fine sliding surfaces, but have little effect on roughness perception for coarse textures. Our behavioural data are consistent with this finding. Discrimination of our coarse gratings was relatively unaffected by the presence or absence of movement. However, discrimination of our fine gratings fell to chance levels when they were statically pressed onto the skin compared with when they were moved across the skin surface (see Roberts et al., 2020 for comparable findings with similar stimuli). Thus, we had anticipated greater overlap in brain activity between vibrotactile stimuli and sliding fine compared with sliding coarse gratings. Our results did support a role for vibration in the processing of touched gratings. Gratings moved across the skin, and therefore likely to generate vibration cues, showed significant overlap with those brain regions activated by vibrotactile stimuli. This contrasted with the static texture conditions, in which vibration cues were expected to be minimal, and which showed no overlap with the areas activated by vibrotactile stimuli. However, we found no significant difference in activation between sliding coarse and fine gratings. This finding raises the possibility that vibration coding is in place as long as there is movement across the skin. It is worth noting that while our main study required participants to monitor the location of the stimuli on the hand rather than the roughness of each surface, a similar pattern of results emerged from our control study where participants attended to surface texture.

Further support for the role of vibration in texture perception comes from our finding that the overlap between vibrotactile regions and those areas stimulated by sliding gratings extended beyond somatosensory to auditory cortex. We are aware of only one previous finding of auditory cortex activity with application of tactile textures in humans. Foxe et al. (2002) examined brain activity associated with the movement of relatively rough (100 grit) sandpaper across the skin. The texture-related activity found in their study overlapped with some of the auditory activation found when their participants simply listened to broadband sounds (modified to approximate the sounds of contact with the sandpaper surface). However, in their study the sandpaper textures were applied using a rolling wheel and so it is unclear whether there would have been sufficient relative motion between stimulus and skin to create skin vibrations of the kind associated with the sliding stimuli used in the present study.

Instead of examining the interactions between auditory and tactile stimuli we sought to determine whether auditory areas contribute to the processing of vibrotactile signals. Auditory activation with our vibrotactile stimuli was not surprising as vibrotactile related activity in auditory regions has been previously been observed (Caetano and Jousmaki, 2006; Schurmann et al., 2006; Nordmark et al., 2012). Our finding that sliding gratings activate some of the same auditory regions as vibrotactile stimuli is novel and strengthens the idea that vibration is a prominent sensory signal during active exploration of gratings. The duplex theory of texture perception predicts that this auditory activity would be more extensive or stronger for fine compared with coarse stimuli. However, as with the other brain areas we found to be activated by both vibrotactile and sliding gratings, the auditory activity related to touch did not change significantly with fine vs. coarse gratings. This may indicate the relative importance of spatial compared with vibration cues for the processing of coarse gratings, with the latter being present but less critical than the former. This might explain why discrimination of coarse gratings is unaffected by vibrotactile adaptation and yet we find evidence of the processing of vibration cues for sliding coarse gratings.

We attribute the differential activation of vibration selective regions by sliding gratings to the role of vibration in the perception of the grating sliding across the index finger. However, it might be argued that a sliding contact constitutes a more salient touch stimulus than a static contact. If higher salience resulted in increased generalized activation, this might explain the difference between sliding and static conditions. We therefore ran a control study in which the static condition was modified to include a double touch. Although this would be expected to increase the salience of the static relative to the sliding condition, we found the contrast between sliding and static touch was maintained and so discount the salience interpretation.

Tactile stimulation of brain areas that are traditionally considered non-somatosensory might not be restricted to auditory cortex. Using tDCS to facilitate performance, Yau et al. (2014) showed the involvement of auditory cortex in processing temporal frequency properties of vibrotactile stimuli. They were also able to demonstrate the involvement of visual cortex in a spatial tactile task (orientation discrimination for tactile gratings). In our study we found auditory activation for tactile stimulation involving sliding but not static gratings. Although we did not find visual activation for static gratings, our stimuli did not include a contrast in terms of spatial properties and our design did not contrast two different tasks.

In finding considerable overlap between areas activated by vibrotactile stimuli and gratings moved across the skin, our work has confirmed the importance of vibration cues in the perception of textures during sliding touch. Furthermore, the activation we found in auditory cortex is consistent with the proposal (Nordmark et al., 2012; Yau et al., 2014) that areas previously considered as unisensory, may contribute to processing of particular features whose sensory content is represented in cortical regions normally identified with another sensory channel.

On the basis of our results it is interesting to ask whether auditory cortex may work as a supramodal processor of the vibratory content of sensory signals originating in auditory or touch pathways? Might the auditory cortex be involved in analysis of the frequency of the vibrotactile stimulus or the spread of frequencies associated with the vibrations arising from the moving tactile grating? In behavioural studies involving frequency judgments, Yau and colleagues have shown intersensory auditory-vibrotactile perceptual biases in both low flutter (around 25 Hz; Convento et al., 2019) and higher frequency (above 100 Hz; Yau et al., 2009, 2010) ranges. Moreover, using fMRI, they went on to demonstrate underlying cross-modal auditory and vibrotactile frequency representations (Rahman et al., 2020). These studies show auditory processing of somatosensory input involving vibrotactile stimulation. Our results indicate auditory cortex is activated by sliding contact with spatial gratings in the same region that is activated by vibrotactile input. What evidence is there to suggest sliding contact might involve a frequency representation of vibration arising from the sliding contact? Evidence of time-based coding of texture has been provided by Long et al. (2022) who showed temporal spiking patterns in primate somatosensory cortex neurons allow decoding of the identity of textures scanned across the finger tips. They also showed that human texture perception is better predicted when spike timing is taken into account. In conclusion, we propose that the auditory cortex activation evident with sliding gratings represents processing of vibratory content of textured tactile input similar to that associated with auditory cortex activation by vibrotactile input.

## Data availability statement

The datasets presented in this study can be found in online repositories. The names of the repository/repositories and accession number(s) can be found below: OSF repository: https://osf.io/eqgr9/?view_only=a67702b38c2147fc8d3ce0b7c7961f2c.

## Ethics statement

The studies involving humans were approved by Research Ethics Team, University of Birmingham. The studies were conducted in accordance with the local legislation and institutional requirements. The participants provided their written informed consent to participate in this study.

## Author contributions

RR: Conceptualization, Data curation, Formal analysis, Funding acquisition, Investigation, Methodology, Project administration, Software, Supervision, Writing – original draft, Writing – review & editing. AL: Data curation, Formal analysis, Investigation, Project administration, Writing – review & editing. HK: Conceptualization, Data curation, Formal analysis, Investigation, Methodology, Project administration, Software, Supervision, Writing – original draft, Writing – review & editing. AW: Conceptualization, Formal analysis, Funding acquisition, Investigation, Resources, Supervision, Visualization, Writing – original draft, Writing – review & editing. HA: Conceptualization, Formal analysis, Funding acquisition, Investigation, Methodology, Resources, Software, Supervision, Validation, Visualization, Writing – original draft, Writing – review & editing.

## Footnotes

^1^ http://www.quaerosys.com

^2^ http://www.fmrib.ox.ac.uk/fsl/index.html
